# Ozone Nanobubble Water as a Sustainable Strategy to Enhance Metabolism, Muscle Function, and Exercise Performance in Mice

**DOI:** 10.3390/nu17172821

**Published:** 2025-08-29

**Authors:** Cheng-Jeng Tsai, Peng-Cheng Hsu, Meng-l Kuo, Yi-Ming Chen

**Affiliations:** 1 Department of Food Science, Fu Jen Catholic University, New Taipei City 242062, Taiwan; 412396043@m365.fju.edu.tw (C.-J.T.); 062998@mail.fju.edu.tw (M.-l.K.); 2 Department of Physical Education, Fu Jen Catholic University, New Taipei City 242062, Taiwan; 154068@mail.fju.edu.tw; 3 Ph.D. Program in Nutrition and Food Science, Fu Jen Catholic University, New Taipei City 242062, Taiwan; 4 College of Human Ecology, Fu Jen Catholic University, New Taipei City 242062, Taiwan

**Keywords:** sustainable sports nutrition, ozone nanobubble water, exercise performance, non-pharmacological intervention, respiratory quotient (RQ)

## Abstract

**Background/Objectives**: Nanobubble water (NBW) is being studied increasingly for its potential benefits in sports nutrition. This study aimed to evaluate whether supplementation with ozone-enriched NBW (O_3_-NBW) could improve integrated exercise capacity—encompassing endurance performance, muscle strength, and postexercise recovery as well as body composition and metabolic adaptations in mice. **Methods:** Male ICR mice (*n* = 24) were allocated into Control, Air-NBW, or O_3_-NBW (0.2–1 mg/L ozone) groups for 4 weeks. **Results:** O_3_-NBW treatment considerably enhanced forelimb grip strength and treadmill running endurance compared to the Control group (both *p* < 0.05). Analyses of body composition revealed a higher proportion of lean mass and muscle glycogen storage in NBW groups, notably with O_3_-NBW. Serum markers gathered post-exercise demonstrated a reduction in ammonia and blood urea nitrogen (BUN), suggesting improved nitrogen metabolism. Levels of resting serum creatine kinase (CK) and uric acid were also lower in O_3_-NBW mice, indicating potential benefits for muscle recovery. In addition, O_3_-NBW treatment significantly enhanced oxygen consumption (VO_2_) and reduced the respiratory quotient (RQ), signifying amplified fat oxidation, while also lowering total energy expenditure (all *p* < 0.05). Spontaneous wheel-running activity remained consistent across all the groups. **Conclusions:** Taken as a whole, these findings emphasize that O_3_-NBW supplementation offers ergogenic and metabolic advantages by improving integrated exercise capacity and efficiency of gas exchange, without adverse effects.

## 1. Introduction

Adequate water supplementation plays a vital role in maintaining physiological homeostasis and optimizing exercise performance. However, the physiological and metabolic implications of water characterized by distinct physicochemical properties remain underexplored. In recent years, nanobubble water (NBW) has emerged as a promising intervention in the fields of sports nutrition and systemic physiological regulation. NBW is distinguished by its nanoscale bubble stability, negatively charged surfaces, elevated gas solubility, and enhanced molecular mobility [1,2]. Due to their minute size (<1 µm) and negative surface charge, nanobubbles can remain stably suspended in solution for extended durations, potentially facilitating mass transfer and modifying localized microenvironments [3,4,5]. These properties have been associated with diverse biological benefits, including the stimulation of plant growth [6,7,8], enhancement of aquaculture yields [9], and improvement of mammalian physiological and metabolic functions [9,10].

In animal studies, oral administration of oxygen nanobubble water has been shown to significantly increase body weight and length in mice, potentially through improved oxygen transport and utilization [9]. Further evidence from alternative biological models suggests that NBW may also support redox homeostasis, accelerate tissue repair, and inhibit pathogenic infection [11,12]. In the context of exercise physiology, these effects translate to potential improvements in aerobic metabolic efficiency, mitigation of tissue hypoxia, and facilitation of lactate and inflammatory mediator clearance—factors that collectively enhance endurance and athletic performance. Moreover, NBWs infused with gases such as hydrogen or nitrogen have demonstrated antioxidant, hepatoprotective, and metabolic-regulating properties in preclinical models [13,14], further underscoring their potential utility as performance-enhancing agents.

Nanobubbles—gas-filled vesicles with diameters below 1 µm—exhibit unique physicochemical characteristics, including high zeta potential and diminished buoyancy, contributing to their exceptional stability in aqueous media [15]. These features enable NBW to maintain supersaturated gas concentrations (e.g., oxygen, ozone), making it advantageous in applications requiring efficient gas delivery [16]. Among these, oxygen and ozone NBW have attracted attention for their potential health and performance-enhancing properties [17,18,19].

Recent studies highlight ozone’s (O_3_) broad applications in medicine, food science, biotechnology, and exercise physiology. In medicine, low-dose ozone acts as a redox bioregulator, enhancing antioxidant defenses, improving oxygen utilization, and reducing inflammation. In food science, ozone water safely extends shelf-life and preserves nutritional quality by inactivating pathogens without chemical residues [20]. In biotechnology, micro/nano ozone bubbles improve water quality, promote plant growth, and serve as eco-friendly disinfectants [21]. In exercise physiology, ozone-based interventions may enhance endurance, muscle recovery, and oxygen delivery, offering a sustainable, non-pharmacological strategy for performance support [22,23]. Ozone (O_3_), in particular, presents a dichotomous effect profile with regard to exercise outcomes, depending on exposure level and delivery method. While high environmental ozone levels can impair pulmonary function and shift metabolism toward inefficient anaerobic pathways thereby reducing endurance [24] controlled low dose ozone therapy has shown ergogenic promise. For example, a controlled study involving athletes revealed that ozone autohemotherapy (20–30 µg/mL, twice weekly for eight weeks) significantly increased VO_2_ max by 28% compared to controls, suggesting enhanced aerobic capacity beyond the effects of standard training [25]. Similarly, in animal models, low-dose ozone treatment has been shown to improve endurance, delay fatigue onset, and reduce markers of muscle damage (e.g., LDH and CK) [19]. Studies in equine athletes have also indicated preserved training adaptations, potentially through hormetic antioxidant mechanisms. Nevertheless, the use of ozone in sports remains controversial. While ozone itself is not listed as a prohibited substance by the World Anti-Doping Agency (WADA), specific methods of administration—especially those involving autologous blood manipulation—are banned [26].

Despite these promising findings, most existing studies have focused on NBW applications in agriculture and biomedicine. Research addressing its role in exercise physiology remains limited, particularly in relation to mechanisms underpinning endurance enhancement, fatigue regulation, and metabolic adaptation. To bridge this gap, the present study investigates whether four weeks of combined NBW and ozone (NBW + O_3_) supplementation can improve body composition, muscle strength, endurance performance, and biochemical fatigue markers in mice. Additionally, we evaluated glycogen storage in liver and skeletal muscle, alongside serum biomarkers indicative of exercise-induced stress and metabolic load. The primary outcome of this study is integrated exercise capacity, defined as the combined assessment of endurance performance, muscle strength, and postexercise recovery, with secondary outcomes including changes in body composition, metabolic adaptations, and biochemical fatigue indices. These analyses aim to provide a comprehensive understanding of the potential ergogenic and metabolic benefits of NBW + O_3_ supplementation within the framework of sustainable, non-pharmacological strategies for performance enhancement.

## 2. Materials and Methods

### 2.1. Prepare of NBW

Nanobubbles (NBs) were generated using a controllable high-pressure platform designed to subject water to high-speed grinding and shearing in the presence of a 3000–4000 E magnetic field (Leading Auto. Bio Co., Ltd., Hsinchu, Taiwan). Under these high-shear conditions, submicron-sized bubbles are produced. For the NBW + O_3_ group, the ozone concentration (0.2–1 mg/L) was verified daily via an iodometric titration method and further measured using a portable dissolved ozone meter (OZ-21P, DKK-TOA Corporation, Tokyo, Japan). Using nanobubble technology can maintain ozone levels of approximately 0.2–1.0 mg/L for extended periods, enhancing both gas solubility and stability in water.

### 2.2. Animals

Male ICR mice, aged 6 weeks, were procured from a certified animal breeding center (BioLASCO, Taipei, Taiwan), and brought to reside in a facility controlled for temperature (24 ± 2 °C) and humidity (60 ± 5%). This facility retained a 12 h light-dark cycle, with a light period from 8:00 to 20:00. The mice subsisted on standard laboratory chow (5001, PMI Feeds) and water, which was made available ad libitum. Following a 2-week acclimatization period, the 24 mice (each roughly 25 g in body weight) were randomly split into three groups, with each group consisting of eight mice. All experimental procedures adhered to the guidelines conducted in strict accordance with the 3R principles

(i) Replacement—We evaluated possible in vitro alternatives; however, a murine model was essential to capture systemic physiological responses.
(ii) Reduction—The number of animals was minimized (*n* = 8 per group) based on sample size calculations to achieve sufficient statistical power.
(iii) Refinement—All procedures were approved by the I Institutional Animal Care and Use Committee (IACUC) of Fu Jen Catholic University, in New Taipei City, Taiwan, and every effort was made to minimize pain, stress, and discomfort. The associated protocol identification number is A11148.

Measures were taken to minimize discomfort amongst the animals, and each mouse was monitored daily for indications of stress or detrimental health effects. Mice were assigned to the following groups for a 4-week intervention: (1) Control (Vehicle): Oral administration of 1 mL distilled water, three times per day. (2) NBW + Air: Oral administration of 1 mL nano-/ultrafine-bubble water (air—NBW + Air), three times daily. (3) NBW + O_3_: Oral administration of 1 mL ozone nanobubble water (0.2–1 mg/L ozone), three times daily. Mice were orally administered 1 mL of the assigned fluid (via gavage) at 07:00, 13:00, and 19:00 for 28 consecutive days. Food intake and body weight were monitored weekly.

### 2.3. Forelimb Grip Strength

On Day 29, the forelimb grip strength of the mice was assessed using a grip strength meter (PicoScope 2000, Pico Technology Limited, Cambridgeshire, UK) 30 min post final gavage. Each mouse was gently made to grasp the metal bar with its forepaws and was then pulled backward until it let go of the bar. The peak force, recorded in grams, was documented. Three trials were conducted for each mouse with resting intervals of 60 s between trials. For analysis, the highest value was used.

### 2.4. Biochemical Indices Related to Fatigue After a 15-Minute Swimming Test

Immediately after 15 min of moderate swimming exercise on Day 29, the mice were placed in water that was 27 ± 1 °C, with a depth of 65 cm and a diameter of 45 cm, for 15 min. Blood samples were collected immediately after exercise to measure lactate, ammonia, BUN, and CK. An additional 0.3 mL blood sample was collected to assess markers of exercise-induced fatigue. These biochemical parameters were analyzed with an automated clinical chemistry analyzer (Hitachi 7060, Tokyo, Japan).

### 2.5. Endurance Performance (Treadmill Exhaustion Test)

On Day 30, we subjected the mice to a treadmill running test (Bio-Cando Incorporation, Taipei, Taiwan) to evaluate their time to exhaustion. Before the test, the mice underwent a 1-week adaptation period to the treadmill, with 10 to 15 min sessions at 10 m/min. On the day of the test, the mice began running on a motorized treadmill set at a 5° incline and a starting speed of 10 m/min. This occurred 30 min after oral gavage. The speed was incrementally increased by 2 m/min every 2 min until reaching 20 m/min. Upon reaching this speed, the mice continued to run until exhaustion. We defined exhaustion as the point at which the mice were unable to maintain running speed despite gentle stimuli, such as a tap or air puff, for 10 s. Both the total running time in minutes and the total distance in meters were recorded.

### 2.6. Blood Collection and Serum Preparation

On Day 31, 24 h after the exhaustion test, the mice were briefly anesthetized. Approximately 0.3 mL of blood was collected from each mouse’s tail vein. The serum was separated via centrifugation at 3000× *g* for 15 min, conducted at 4 °C, and was then stored at –80 °C until it was time for analysis. The blood sample was collected aspartate aminotransferase (AST), alanine aminotransferase (ALT), alkaline phosphatase (ALP), lactate dehydrogenase (LDH), creatinine, albumin, total protein (TP), Uric acid, total cholesterol (TC), high-density lipoprotein (HDL), low-density lipoprotein (LDL), triacylglycerol (TG), glucose, creatine kinase (CK). These biochemical parameters were analyzed with an automated clinical chemistry analyzer (Hitachi 7060, Tokyo, Japan).

### 2.7. Tissue Collection and Glycogen Analysis

All mice were euthanized 30 min after the final gavage (Day 31). Under anesthesia, the liver and the hindlimb skeletal muscle (gastrocnemius) were promptly removed, rinsed with cold saline, blotted dry, and weighed. The tissues were then frozen in liquid nitrogen and stored at −80 °C for later analysis. The phenol-sulfuric acid method was employed to assess tissue glycogen content. To summarize the process, samples (100 mg) were homogenized in five volumes of homogenization buffer using a Bullet Blender (Next Advance, Troy, NY, USA), then centrifuged at 12,000× *g* for 15 min at 4 °C. The collected supernatant was mixed with anthrone reagent. The glycogen concentration was calculated by comparing the absorbance at 620 nm against a standard curve generated from purified glycogen (Sigma-Aldrich, St. Louis, MO, USA).

### 2.8. Body Composition Measurement

A time-domain nuclear magnetic resonance (TD-NMR) instrument (Minispec LF50, Bruker, Billerica, MA, USA) was employed to evaluate in vivo body composition (fat mass, lean mass, and fluid content) in conscious mice. Observations were made at baseline and the culmination of the 4-week intervention. The instrument was calibrated per the manufacturer’s guidelines, utilizing a 0.17 T magnetic field that corresponds to a proton frequency of 7.5 MHz. Body composition indices were denoted as a percentage of total body weight for fat and lean mass.

### 2.9. Measuring Energy Metabolism and Spontaneous Physical Activity

After 4 weeks of NBW supplementation, the mice were housed individually in an energy-metabolism system for 5 days. During this period, they had unlimited access to food and water. Respiratory gases, including water vapor, were measured using an integrated system. This system consisted of a fuel cell oxygen analyzer, a spectrophotometric carbon dioxide analyzer, and a capacitive water vapor partial pressure analyzer. The respiratory quotient (RQ) was calculated as the ratio of CO_2_ production to O_2_ consumption. Energy expenditure (EE) was determined using the Weir equation: 60 × [0.003941 × oxygen consumption (VO_2_) + 0.001106 × carbon dioxide production (VCO_2_)]. All metabolic cages were outfitted with running wheels to continuously measure spontaneous physical activity (SPA) over 5 days. Total wheel revolutions were recorded daily, and the total distance run per day was determined by multiplying the number of wheel rotations by the wheel’s circumference. This arrangement is viewed as a form of voluntary exercise.

### 2.10. Statistical Analysis

All results are presented as the mean ± standard deviation (SD). Statistical analyses were conducted using one-way analysis of variance (ANOVA), followed by Tukey’s HSD test to evaluate differences among the three groups (Control, NBW + Air, NBW + O_3_). A *p*-value of less than 0.05 was considered statistically significant. The software package used for the analysis was SAS (Version 9.4, SAS Institute, Cary, NC, USA). Using G*Power (Version 3.1.9.7, Universität Düsseldorf, Düsseldorf, Germany) (one-way ANOVA, 3 groups, α = 0.05), an a priori analysis shows that *n* = 24 (*n* = 8/group) provides > 0.90 power to detect a conservative large effect (Cohen’s f = 0.40). To assess the magnitude of differences, effect sizes (ES) were calculated using Cohen’s d. For between-group comparisons, Cohen’s d was computed based on pooled standard deviations. The interpretation of d was as follows: small (0.2 ≤ d < 0.5), medium (0.5 ≤ d < 0.8), and large (d ≥ 0.8). Effect sizes were reported alongside *p*-values to provide additional insight into the practical significance of the findings.

## 3. Results

### 3.1. Effects of Air-NBW and O_3_-NBW on Body Weight, Dietary Intake, and Body Composition in Mice

**Figure 1:**
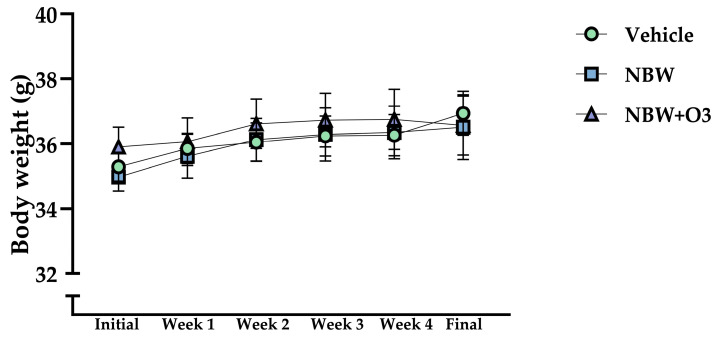
Increased body weight in air-infused nano-bubble water (Air-NBW) or ozone-infused nano-bubble water (O_3_-NBW)-treated mice. Male ICR mice were supplemented with the vehicle, Air-NBW, and O_3_-NBW, for 4 weeks. Values are the mean ± SD for *n* = 8 mice per group.

Over the entire experimental period, body weight steadily increased in all three groups without any significant variations across the groups (Figure 1). Specifically, no notable changes in the final body weight were observed in either the Air-NBW or O_3_-NBW groups compared to the vehicle Control group.

Dietary intake was documented and the summary can be found in Table 1. The analysis demonstrated that supplementation with either Air-NBW or O_3_-NBW did not significantly modify the animals’ daily food consumption when compared with the Control group (*p* > 0.05). Therefore, it seems neither Air-NBW nor O_3_-NBW supplementation affects the appetite or feeding behavior in mice. Thus, neither of these supplements appeared to harm the mice’s normal growth patterns. The data indicate that NBW supplementation resulted in differences in muscle weight (*p* = 0.0014, ES = 1.1767; *p* = 0.0004, ES = 1.9612) and relative muscle weight (*p* = 0.0273, ES = 1.1043; *p* = 0.0009, ES = 1.9403) in the Air-NBW and O_3_-NBW groups when compared with the Control group. Details regarding body composition data were gathered using a mouse body composition analyzer procedure.

**Figure 2:**
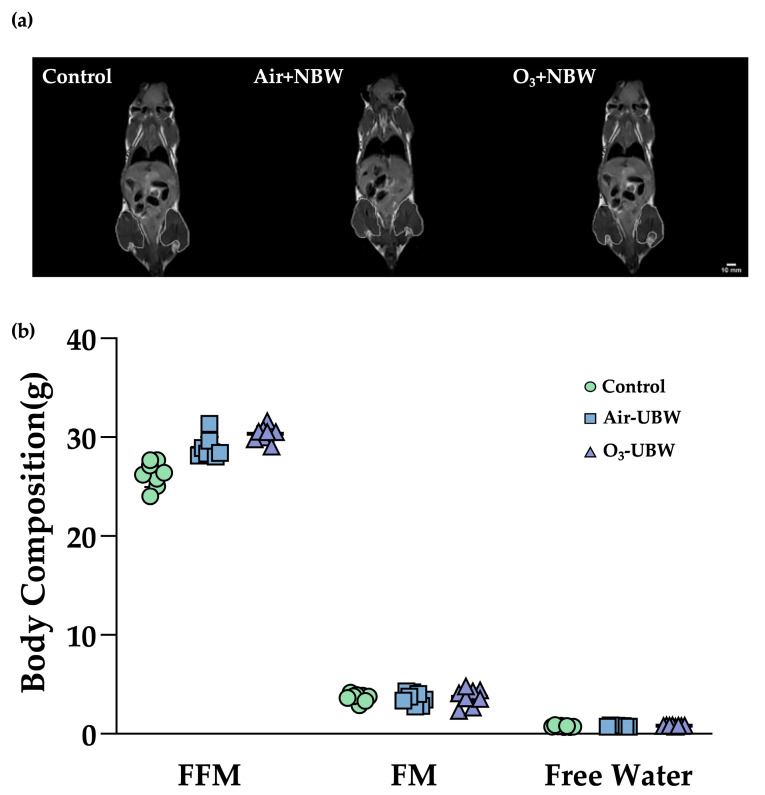
(**a**) MRI examination of mice supplemented with air-infused nano-bubble water (Air-NBW) or ozone-infused nano-bubble water (O_3_-NBW). (**b**) Final measurements were taken 4 weeks after Air-NBW and O_3_-NBW supplementation, where fluid and soft tissues (muscle and fat) were estimated from hydrogen spin signals. Body composition was measured using a Minispec LF50 TD-NMR instrument (Bruker, Ettlingen, Germany), which analyzes hydrogen proton relaxation signals to quantify whole-body fat mass, lean tissue mass, and total body water. Male ICR mice were supplemented with the vehicle, Air-NBW, and O_3_-NBW, for 4 weeks. Values are expressed as mean ± SD (*n* = 8 mice per group).

Body composition was evaluated after the experiment using MRI techniques. As depicted in Figure 2a, a preliminary visual inspection of MRI scans found no notable differences among the groups. To obtain quantitative indices, we estimated fluid and soft tissues (muscle and fat) using hydrogen spin signals. As indicated in Figure 2b, the Free Fat Mass (FFM) values for the Control, Air-NBW, and O_3_-NBW groups were 26.25 ± 1.20 g, 28.92 ± 1.04 g, and 30.35 ± 0.71 g, respectively. The FFM was significantly higher in both the Air + NBW group (1.10-fold, *p* < 0.0001, ES = 2.3779) and the O_3_ + NBW group (1.16-fold, *p* < 0.0001, ES = 4.1585) as compared to the Control group. Fat Mass (FM), in contrast, was 3.65 ± 0.37 g, 3.57 ± 0.55 g, and 3.71 ± 0.81 g for the Control, Air-NBW, and O_3_-NBW mice, respectively, without any significant disparities among the three groups (F(2,23) = 0.09, *p* = 0.9121). Likewise, no significant effects were observed for total body free water among the groups (F(2,23) = 2.03, *p* = 0.1564). This rise in FFM implies that NBW supplementation could aid in the enhancement of lean body mass without affecting FM or fluid balance.

### 3.2. Effects of Air-NBW and O_3_-NBW on Exercise Performance

**Figure 3:**
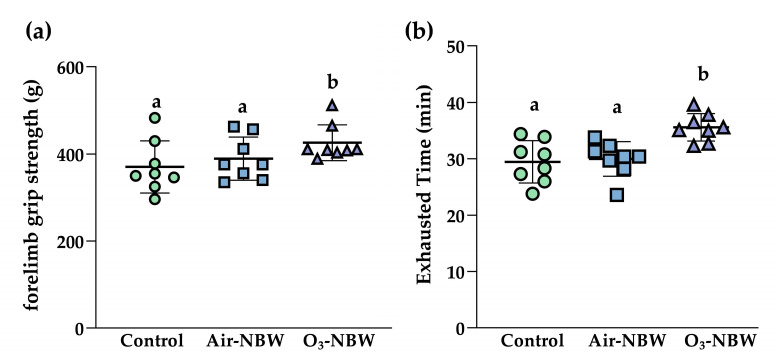
Effect of air-infused nano-bubble water (Air-NBW) or ozone-infused nano-bubble water (O_3_-NBW) supplementation on (**a**) forelimb grip strength and (**b**) exhaustive treadmill test. Values are expressed as mean ± SD (*n* = 8 mice per group). Bars with different superscript letters (^a, b^) indicate significant differences among groups (*p* < 0.05) according to one-way ANOVA followed by Tukey’s HSD post hoc test.

To determine if Air-NBW or O_3_-NBW could boost muscle strength, we performed a forelimb grip strength test after 4 weeks of daily supplementation. Thirty minutes after the final intake on the last day of the experiment, mice were tested using a validated grip strength measuring device in our laboratory. As shown in Figure 3a, the average grip strength (g) for the Control, Air-NBW, and O_3_-NBW groups was 370.4 ± 59.9, 389.4 ± 49.8, and 426.0 ± 41.1 g, respectively. The O_3_-NBW group displayed a significant 1.15-fold increase compared to the Control group (*p* = 0.0400, ES = 1.0824), suggesting that O_3_-NBW has a distinct enhancing effect on mice’s forelimb muscle strength. Despite a slight rise observed in the Air-NBW group, it did not attain statistical significance.

The endurance performance of mice was further analyzed using a treadmill running exhaustion test. After 4 weeks of supplementation, the mice were tested again 30 min following the final administration. The treadmill exhaustion times for the Control, Air-NBW, and O_3_-NBW groups were 29.5 ± 3.8, 30.0 ± 3.1, and 35.6 ± 2.4 min, respectively (Figure 3b). When compared to controls, the O_3_-NBW group showed a 1.21-fold increase in running endurance (*p* = 0.0008, ES = 1.9194). This significant improvement suggests that O_3_-NBW markedly enhances endurance capacity.

### 3.3. Effects of NBW and NBW + O_3_ on Blood Biochemical Parameters Following Exercise Challenge

**Figure 4:**
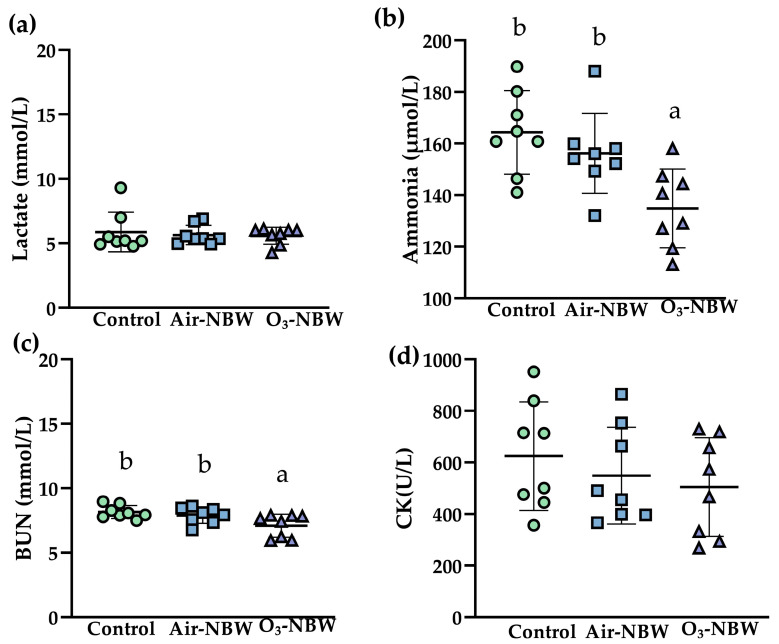
Effect of air-infused nano-bubble water (Air-NBW) or ozone-infused nano-bubble water (O_3_-NBW) supplementation on the serum levels of (**a**) lactate, (**b**) ammonia, (**c**) blood urea nitrogen (BUN), (**d**) creatine kinase (CK), after a 15 min swimming l test. Values are expressed as mean ± SD (*n* = 8 mice per group). Bars with different superscript letters (^a, b^) indicate significant differences among groups (*p* < 0.05) according to one-way ANOVA followed by Tukey’s HSD post hoc test.

After 4 weeks of supplementation, an acute swimming challenge was carried out to further evaluate exercise-related biochemical markers. Specifically, the mice were placed in water that was 27 ± 1 °C, with a depth of 65 cm and a diameter of 45 cm, for 15 min. Blood samples were collected immediately after exercise to measure lactate, ammonia, BUN, and CK, as displayed in Figure 4a–d.

The concentrations of lactate in the Control, Air-NBW, and O_3_-NBW groups were 5.9 ± 1.5, 5.6 ± 0.7, and 5.6 ± 0.7 mmol/L, respectively (Figure 4a). No significant differences were found among the three groups (*p* = 0.8407), suggesting that neither NBW nor O_3_-NBW supplementation affected lactate accumulation under the conditions tested. The ammonia levels were significantly lower in the O_3_-NBW group (134.8 ± 15.3 μmol/L) compared to the Control (164.3 ± 16.2 μmol/L; *p* = 0.0011, ES = 1.8723), reflecting a 17.96% decrease (Figure 4b). This improvement in ammonia clearance suggests that O_3_-NBW could help attenuate muscle protein catabolism or enhance the efficiency of nitrogen metabolism following acute exercise stress.

The O_3_-NBW group also exhibited lower BUN concentrations (7.1 ± 0.9 mmol/L) compared to the Control group (8.2 ± 0.5 mmol/L; *p* = 0.0062, ES = 1.5110), corresponding to a 12.94% reduction (Figure 4c). This further supports the concept that O_3_-NBW supplementation might reduce the burden of nitrogen waste products during or following high-intensity exercise. CK levels measured immediately post-exercise for the Control, Air-NBW, and O_3_-NBW groups were 624.2 ± 210.6, 548.5 ± 187.4, and 504.5 ± 190.9 U/L, respectively (Figure 4d). Even though there was a non-significant trend toward reduced CK in both the Air-NBW and O_3_-NBW groups, statistical analysis showed no significant differences among the groups (*p* = 0.4806). Nevertheless, the slight downward trend in CK might indicate a potential protective effect on muscle integrity, though more sensitive markers or alternative time points might be needed to confirm this. In conclusion, O_3_-NBW supplementation significantly decreased both ammonia and BUN levels in mice after exercise, implying that this intervention might foster a more favorable metabolic environment for exercise recovery.

### 3.4. Effects of NBW and NBW + O_3_ on Final Blood Biochemical Values

After the 4-week supplementation period, blood samples were collected from the mice at the time of sacrifice. These samples were used to evaluate various biochemical parameters (Table 2), including AST (aspartate transaminase), ALT (alanine aminotransferase), ALP (alkaline phosphatase), LDH (lactate dehydrogenase), TP (total protein), TC (total cholesterol), HDL (high-density lipoprotein), LDL (low-density lipoprotein), TG (triacylglycerol), and CK. This profile was employed in the assessment of systemic health and potential toxicity.

Both the Air-NBW and O_3_-NBW-supplemented groups exhibited a notable decline in uric acid levels in comparison with the Control group (F(2,23) = 18.31, *p* < 0.0001, ES = 1.5474 and 3.6375). Significantly, the O_3_-NBW group showed a 32.93% decrease compared to the Control group (*p* < 0.0001, ES = 3.6375), suggesting a systemic impact on purine metabolism or improved uric acid clearance. Serum CK levels also decreased significantly in the NBW and O_3_-NBW groups compared to the Control group (F(2,23) = 22.73, *p* < 0.0001, ES = 3.2940). Even though the reduction in CK was not statistically significant following the exercise challenge, in a resting state, these measurements imply potential benefits in maintaining muscle integrity.

There were no significant changes observed in the Liver and Metabolic Markers, including AST, ALT, ALP, LDH, TP, TC, HDL, LDL, TG, or glucose. These findings suggest that supplementing with NBW or O_3_-NBW for 4 weeks did not induce hepatic toxicity, renal overload, or dyslipidemia, demonstrating a safe profile under the conditions that were tested. Taken together, these results indicate that continuous supplementation with NBW or O_3_-NBW for 4 weeks can lower uric acid and resting CK levels, without any negative impact on liver and metabolic profiles. This provides further evidence of the beneficial role of NBW and O_3_-NBW supplementation in enhancing physiological homeostasis.

### 3.5. Effects of NBW and O_3_-NBW on Hepatic and Muscular Glycogen Stores

**Figure 5:**
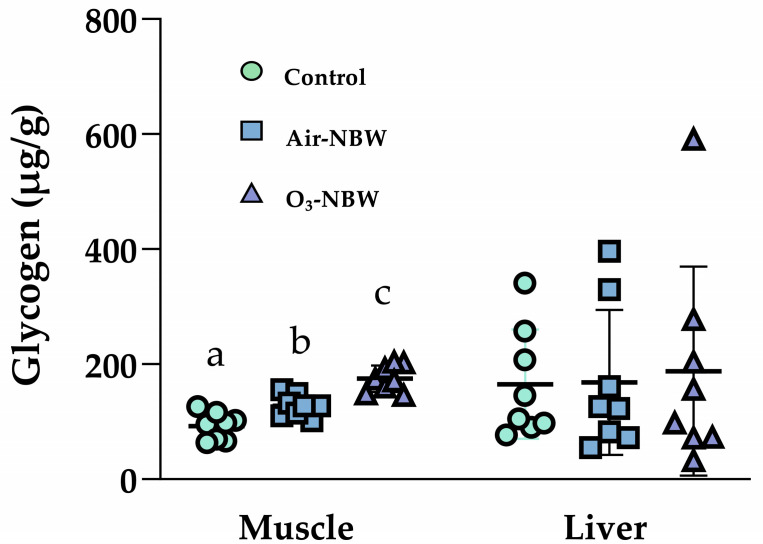
Effect of air-infused nano-bubble water (Air-NBW) or ozone-infused nano-bubble water (O_3_-NBW) supplementation on muscle glycogen and liver glycogen. Values are expressed as mean ± SD (*n* = 8 mice per group). Bars with different superscript letters (^a, b, c^) indicate significant differences among groups (*p* < 0.05) according to one-way ANOVA followed by Tukey’s HSD post hoc test.

To determine whether NBW or O_3_-NBW supplementation increases glycogen storage, we excised hepatic and gastrocnemius muscle tissues 1 h after the final supplementation at the end of the experiment and analyzed them for glycogen content (Figure 5). Muscle glycogen content in the Control, Air-NBW, and O_3_-NBW groups was 92.3 ± 22.0, 127.4 ± 17.3, and 174.7 ± 21.5 μg/g, respectively. Both the Air-NBW and O_3_-NBW supplemented groups showed a significant increase relative to the Control group (1.38-fold, *p* = 0.0041, ES = 1.7736; and 1.89-fold, *p* < 0.0001, ES = 3.7883, respectively).

However, no significant differences were observed in hepatic glycogen content among the three groups (165.3 ± 88.8, 168.2 ± 118.0, and 170.3 ± 168.7 μg/g for Control, Air-NBW, and O_3_-NBW, respectively; F(2,23) = 0.06, *p* = 0.9393). This suggests the beneficial impact of NBW or O_3_-NBW on glycogen storage may be more pronounced in skeletal muscle than in the liver. Increased muscle glycogen availability could partly account for the observed improvements in grip strength and endurance performance, considering muscle glycogen is a vital substrate during prolonged or intense physical activity.

### 3.6. Effect of 4-Week Air-NBW and O_3_-NBW Supplementation on Energy Metabolism

**Figure 6:**
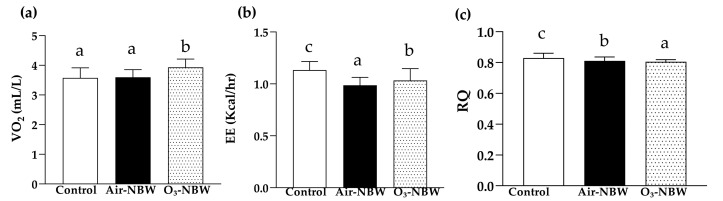
Effect of air-infused nano-bubble water (Air-NBW) or ozone-infused nano-bubble water (O_3_-NBW) supplementation on (**a**) volume of oxygen consumption, (**b**) energy expenditure and (**c**) respiratory quotient. Mice were pretreated with the vehicle, Air-NBW, and O_3_-NBW for 4 weeks. They were measured using respirometric indirect calorimetry over 72 h during the NBW or vehicle infusions. Values are expressed as mean ± SD (*n* = 8 mice per group). Bars with different superscript letters (^a, b, c^) indicate significant differences among groups (*p* < 0.05) according to one-way ANOVA followed by Tukey’s HSD post hoc test.

The average 72 h VO_2_ values for the Control, Air-NBW, and O_3_-NBW groups were 3.57 ± 0.34, 3.60 ± 0.26, and 3.93 ± 0.28 mL/L, respectively (Figure 6a). Notably, O_3_-NBW was significantly higher (1.10-fold, *p* < 0.0001, ES = 1.1559) than the Control group. We measured EE using respirometric indirect calorimetry over 72 h during Control, Air-NBW, and O_3_-NBW infusions. To ascertain the effect of EE, we measured VCO_2_ and VO_2_ to compute the RQ. This quotient is utilized to estimate the predominant fuel source [27]. The average EE in the Control, Air-NBW, and O_3_-NBW groups was 1.13 ± 0.08, 0.99 ± 0.08, and 1.03 ± 0.11 kcal/h, respectively (Figure 6b). EE in the Air-NBW and O_3_-NBW groups was significantly lower, by 12.87% (*p* < 0.0001, ES = 1.7500) and 8.82% (*p* < 0.0001, ES = 1.0398), respectively, compared with the Control group. The RQ of the Control, Air-NBW, and O_3_-NBW groups was 0.83 ± 0.03, 0.81 ± 0.02, and 0.80 ± 0.01 (VCO_2_/VO_2_), respectively (Figure 6c). When compared with the Control group, RQ was significantly decreased by 2.19% (*p* < 0.0001, ES = 0.7845) in the Air-NBW group and by 2.97% (*p* < 0.0001, ES = 1.3416) in the O_3_-NBW group. We also evaluated in-cage SPA. The total distance covered in wheel running did not differ significantly among the groups. Our results indicate that O_3_-NBW infusion significantly raises VO_2_ while decreasing EE and RQ in comparison to the Control group. Air-NBW also reduces EE and slightly lowers RQ. However, no significant differences in SPA (as measured by wheel running) were observed across the groups.

## 4. Discussion

Ozone therapy has garnered attention in sports medicine due to its potential benefits in accelerating recovery and controlling inflammation [28]. It is suggested that ozone can aid muscle repair by enhancing oxygen utilization and cellular metabolism [29]. Animal model studies propose that ozone therapy may potentially stimulate stem cell activation, thereby expediting muscle regeneration and repair after rigorous exercise [19]. As a result, ozone-based medical gases could help mitigate the risk of chronic fatigue in athletes [30]. However, this study only offers provisional data and potential mechanistic insights. The application of ozone therapy in athletes requires thoughtful navigation of anti-doping regulations and related ethical guidelines.

Recent studies on both ozone therapy and nanobubble water report benefits that closely mirror our findings in terms of enhanced antioxidant capacity, improved metabolic regulation, and better exercise performance. For example, one study found that mice given ozone-treated pitaya polyphenols had significantly longer endurance (increase in exhaustion time) along with a higher total antioxidant capacity and markedly greater liver glycogen stores compared to controls [31]. These outcomes suggest that ozone exposure can bolster antioxidant defenses and energy substrate availability during exercise. Similarly, nanobubble water has demonstrated anti-fatigue and metabolic benefits in animal models: one study showed that rats drinking nanobubble rich water swam longer and had lower blood lactate, while exhibiting elevated muscle hemoglobin and higher antioxidant enzyme activities alongside reduced markers of oxidative damage. Notably, their liver glycogen was also significantly increased, indicating improved fuel conservation [32]. Such enhancements in antioxidant status and energy metabolism parallel the lower respiratory quotient (RQ) and higher glycogen levels we observed, reflecting a shift toward fat oxidation and sparing of carbohydrates. Even in human trials, oxygenated nanobubble beverages have shown ergogenic effects improving cycling endurance and power output in athletes [18]. Collectively, these studies corroborate that moderate ozone or nanobubble interventions can heighten antioxidant capacity, favor fat-based metabolism, and ultimately improve performance during exercise, which is consistent with the metabolic profile induced by O_3_-NBW in our study.

Our study showed that O_3_-NBW increases VO_2_ while reducing both the RQ and total EE. These effects arise from a combination of acute oxidative stress and subsequent mitochondrial adaptations. By enhancing the solubility and stability of ozone [33], NBW technology intensifies the oxidative signals responsible for these metabolic shifts.

In the initial phase, ozone-induced oxidative stress triggers neurohormonal responses—primarily glucocorticoid and catecholamine release—which amplify fat oxidation (evident by a lower RQ) and induce a transient hypometabolic state that decreases EE [34]. Concurrently, the presence of nanobubbles could stimulate the generation of reactive oxygen species (ROS) by the heightened rate of ozone dissolution, and potentially enable ozone-water interactions at the gas–liquid interface [35].

Elevated ROS levels may sabotage mitochondrial efficiency, inducing the organism to consume more oxygen (VO_2_) to maintain ATP production, even though the total EE remains reduced. Over time, adaptive (hormetic) mechanisms predominate. Thus, through enhanced ozone solubility and stability, NBW proposes a more consistent oxidative stimulus that reinforces these responses. Ultimately, O_3_-NBW nurtures a metabolic state characterized by greater reliance on fat oxidation, increased oxygen usage, and reduced overall EE. When an organism elevates its VO_2_ and predominantly relies on fat oxidation, it can maintain a stable ATP supply without excessively depleting its glycogen or amino acids [36]. Our data align with this concept, indicating that both the Air-NBW and O_3_-NBW groups exhibit higher muscle glycogen levels compared to the Control group. Additionally, the Air-NBW and O_3_-NBW treatments lead to decreased ammonia post-acute exercise, implying lesser protein catabolism under such conditions. Even though initial oxidative stress might temporarily impair mitochondrial efficiency, consistent or repeated exposure can eventually enhance mitochondrial function [37]. This adaptive response, along with increased antioxidant defenses, aids in preserving muscle protein and supports the maintenance or even enhancement of muscle mass [38].

The convergence of findings above points to common mechanistic underpinnings involving mitochondrial function and oxidative stress modulation. Ozone therapy at low doses is known to act as a mild eustress, provoking a transient oxidative stimulus that paradoxically leads to stronger antioxidant defenses. Mechanistically, ozone readily dissolves and reacts to form reactive oxygen species like H_2_O_2_, which in turn activate the Nrf2 pathway a master regulator of antioxidant responses [39]. This results in up-regulation of endogenous antioxidants, fortifying the cell’s capacity to neutralize exercise-induced ROS. In our context, the O_3_-NBW likely triggers a similar hormetic response: an initial burst of ROS signals stimulates protective pathways before being quenched by the enhanced antioxidant machinery. This controlled oxidative stress can also influence mitochondrial efficiency. Recent work by Oliveira et al. (2024) showed that short-term ozone treatment in mice mildly inhibited certain mitochondrial respiratory complexes (I and II/III) without affecting complex IV, thereby limiting excessive ROS production at its source [40,41]. This suggests that mitochondria adapt to the oxidative challenge by becoming more efficient—consuming more oxygen (higher VO_2_) to sustain ATP generation while minimizing oxidative damage. In practical terms, such adaptation would explain why O_3_-NBW-treated animals in our study showed increased oxygen uptake with lower overall energy expenditure: their mitochondria may be working in a “high-oxygen, high-fat oxidation” mode that yields sufficient ATP without wasteful fuel burning. By reducing exercise-induced oxidative stress and improving mitochondrial metabolic flexibility, ozone-infused NBW helps preserve muscle protein and glycogen, as evidenced by the lower ammonia levels and higher postexercise muscle glycogen in treated groups. In summary, the enhanced mitochondrial efficiency and robust antioxidant network induced by O_3_-NBW not only protect against oxidative damage during exercise but also promote a greater reliance on fat for fuel (lower RQ), thereby sustaining performance and aiding recovery. These mechanistic insights underscore O_3_-NBW as a novel, non-invasive strategy for improving metabolic resilience and exercise capacity in athletes, aligning with the broader literature on ozone and nanobubble interventions in sports science. Above all, the use of NBW, particularly when paired with low-dose ozone, represents a novel, non-invasive, and potentially scalable strategy to support athletic recovery and performance enhancement.

Limitation: This preclinical study has limitations. First, the use of young male ICR mice and a 4-week intervention limits generalizability across sex, strain, age, and longer durations. Second, O_3_-NBW was tested at a single concentration range (0.2–1 mg/L) without dose–response or pharmacokinetic evaluation, and thrice-daily gavage may have induced handling stress. Third, no direct measures of oxidative stress, mitochondrial function, or muscle histology were included. Finally, translational relevance to humans remains uncertain; safety, efficacy, optimal dosing, delivery mode, and compliance with anti-doping regulations require confirmation in well-designed clinical trials.

## 5. Conclusions

Our results indicate that both Air-NBW and O_3_-NBW improve muscle mass and exercise-related biochemical markers in mice. Notably, O_3_-NBW shows greater benefits in endurance, strength, and post-exercise recovery. These findings suggest that NBW, especially when combined with ozone, offers a sustainable, non-pharmacological approach to enhancing physical performance while supporting long-term physiological health. Given these promising results, O_3_-NBW may hold translational potential as a sports nutrition supplement to enhance performance and recovery in humans. However, its clinical application requires confirmation through rigorously designed trials to establish efficacy, safety, optimal dosing, and delivery strategies, while ensuring compliance with ethical standards and anti-doping regulations.

## Figures and Tables

**Table 1:** Body weight, food and water intake, and postmortem organ weights of male ICR mice supplemented with vehicle, Air-NBW, or O_3_-NBW for 4 weeks.

Characteristics	Control	Air + NBW	O_3_ + NBW	*p*-Value
Initial BW (g)	35.29 ± 1.27	34.98 ± 1.23	35.90 ± 1.72	0.4329
Final BW (g)	36.94 ± 1.92	36.51 ± 2.82	36.56 ± 2.57	0.9317
Food intake (g/day)	8.00 ± 1.32	8.01 ± 1.14	8.05 ± 2.01	0.9939
Water intake (mL/day)	4.05 ± 0.83	4.06 ± 0.56	4.06 ± 0.59	0.9493
Postmortem organ weights (g)				
Liver (g)	1.51 ± 0.13	1.50 ± 0.10	1.51 ± 0.10	0.9736
Kidney (g)	0.26 ± 0.02	0.25 ± 0.02	0.25 ± 0.02	0.3508
Lung (g)	0.21 ± 0.01	0.21 ± 0.01	0.22 ± 0.01	0.5970
EFP (g)	0.60 ± 0.19	0.54 ± 0.09	0.52 ± 0.08	0.5144
Muscle (g)	0.33 ± 0.03 ^a^	0.36 ± 0.02 ^b^	0.38 ± 0.02 ^b^	0.0014
Heart (g)	0.18 ± 0.02	0.18 ± 0.02	0.18 ± 0.01	0.8276
Relative liver weight (%)	4.08 ± 0.28	4.12 ± 0.39	4.15 ± 0.37	0.9224
Relative Kidney weight (%)	0.70 ± 0.07	0.68 ± 0.08	0.69 ± 0.08	0.8595
Relative Lung weight (%)	0.58 ± 0.02	0.58 ± 0.07	0.59 ± 0.06	0.8595
Relative EFP weight (%)	1.62 ± 0.53	1.47 ± 0.22	1.43 ± 0.23	0.5409
Relative Muscle weight (%)	0.89 ± 0.10 ^a^	0.99 ± 0.08 ^b^	1.05 ± 0.06 ^b^	0.0032
Relative Heart weight (%)	0.46 ± 0.10	0.49 ± 0.06	0.62 ± 0.06	0.9755

Data is presented as mean ± SD, *n* = 8 mice/group. Values are presented as mean ± SD (*n* = 8 per group). Different superscript letters (^a, b^) denote statistically significant differences between groups (*p* < 0.05), as determined by one-way ANOVA followed by Tukey’s HSD post hoc test. Muscle mass includes both gastrocnemius and soleus muscles at the back part of the lower legs. BW, body weight; EFP, epididymal fat pad. The mice were randomly assigned to receive either vehicle (distilled water), Air + NBW, or O_3_ + NBW (ozone concentration of 0.2–1 mg/L). Each group was given 1 mL of the designated fluid by oral gavage at 07:00, 13:00, and 19:00 daily for 28 consecutive days.

**Table 2:** Effect of 4-week Air-NBW and O_3_-NBW supplementation on biochemical variables.

Parameter	Control	Air + NBW	O_3_ + NBW	*p*-Value
AST (U/L)	51.1± 22.2	49.3 ± 14.0	48.3 ± 8.1	0.8936
ALT (U/L)	76.9 ± 32.4	68.6 ± 26.6	59.8 ± 16.0	0.4775
ALP (U/L)	92.3 ± 22.2	94.9 ± 13.8	95.0 ± 23.9	0.9606
LDH (U/L)	790 ± 105	786 ± 84	815 ± 75	0.8028
Creatinine (μmol/L)	74.9 ± 3.1	80.1 ± 2.9	81.7 ± 3.2	0.9081
Albumin (g/L)	31.4 ± 1.4	32.0 ± 1.8	32.5 ± 1.3	0.4008
TP (g/L)	50.0 ± 2.1	50.6 ± 2.9	51.0 ± 3.9	0.8217
Uric acid (μmol/L)	266 ± 35	331 ± 48	397 ± 37	<0.0010
TC (mg/dL)	155 ± 32	146 ± 25	143 ± 15	0.6458
HDL (mg/dL)	144 ± 23	146 ± 19	149 ± 14	0.9081
LDL (mg/dL)	26 ± 11	22 ± 9	22 ± 7	0.5975
TG (mg/dL)	166 ± 29	151 ± 29	149 ± 31	0.4845
Glucose (mg/dL)	100 ± 16	104 ± 19	105 ±24	0.8233
CK (U/L)	493 ± 129 ^c^	360 ± 71 ^b^	174 ± 46 ^a^	<0.0010

Data is expressed as mean ± SD, *n* = 8 mice/group. Values are presented as mean ± SD (*n* = 8 per group). Different superscript letters (^a, b, c^) denote statistically significant differences between groups (*p* < 0.05), as determined by one-way ANOVA followed by Tukey’s HSD post hoc test. AST, aspartate aminotransferase; ALT, alanine aminotransferase; ALP, alkaline phosphatase; LDH, lactate dehydrogenase; TP, total protein; TC, total cholesterol; HDL, high-density lipoprotein; LDL, low-density lipoprotein; TG, triacylglycerol; CK, creatine kinase.

## Data Availability

Summary data supporting the findings of this study are available within the article. Raw datasets are available from the corresponding author upon reasonable request.

## Abbreviations

The following abbreviations are used in this manuscript:

NBW	Nanobubble Water
O_3_-NBW	Ozone-infused Nanobubble Water
Air-NBW	Air-infused Nanobubble Water
VO_2_	Oxygen Consumption
RQ	Respiratory Quotient
EE	Energy Expenditure
CK	Creatine Kinase
BUN	Blood Urea Nitrogen
LDH	Lactate Dehydrogenase
AST	Aspartate Transaminase
ALT	Alanine Aminotransferase
ALP	Alkaline Phosphatase
TP	Total Protein
TC	Total Cholesterol
HDL	High-Density Lipoprotein
LDL	Low-Density Lipoprotein
TG	Triacylglycerol
SPA	Spontaneous Physical Activity
FFM	Free Fat Mass
FM	Fat Mass
EFP	Epididymal Fat Pad
ICR	Institute of Cancer Research (mice strain)
NMR	Nuclear Magnetic Resonance
MRI	Magnetic Resonance Imaging
